# Increased Sensitivity of *Plasmodium falciparum* to Artesunate/Amodiaquine Despite 14 Years as First-Line Malaria Treatment, Zanzibar

**DOI:** 10.3201/eid2608.191547

**Published:** 2020-08

**Authors:** Mwinyi Msellem, Ulrika Morris, Aungpaing Soe, Faiza B. Abbas, Abdul-Wahid Ali, Rory Barnes, Paolo Frumento, Abdullah S. Ali, Andreas Mårtensson, Anders Björkman

**Affiliations:** Mnazi Mmoja Hospital, Zanzibar, Tanzania (M. Msellem);; Karolinska Institutet, Stockholm, Sweden (U. Morris, A. Soe, R. Barnes, P. Frumento, A. Bjorkman);; Zanzibar Malaria Elimination Programme, Zanzibar, Tanzania (F.B. Abbas, A.-W. Ali, A.S. Ali);; Uppsala University, Uppsala, Sweden (A. Mårtensson)

**Keywords:** Plasmodium falciparum, parasites, malaria, drug resistance, genotypes, resistance selection, *P. falciparum* chloroquine-resistance transporter gene, *pfcrt* gene, *P. falciparum* multidrug-resistance 1 gene, *pfmdr1* gene, *P. falciparum* Kelch 13 propeller domain gene, *pfk13* gene, trends, artemisinin-based combination therapy, ACT, artesunate/amodiaquine, ASAQ, vector-borne infections, zoonoses, Zanzibar, Tanzania

## Abstract

Artemisinin-based combination therapies (ACTs) are first-line treatments for uncomplicated *Plasmodium falciparum* malaria. ACT resistance is spreading in Asia but not yet in Africa. Reduced effects of ACT partner drugs have been reported but with little information regarding widely used artesunate/amodiaquine (ASAQ). We studied its efficacy in Zanzibar after 14 years as first-line treatment directly by an in vivo, single-armed trial and indirectly by prevalences of different genotypes in the *P. falciparum* chloroquine-resistance transporter, multidrug-resistance 1, and Kelch 13 propeller domain genes. In vivo efficacy was higher during 2017 (100%; 95% CI 97.4%–100%) than during 2002–2005 (94.7%; 95% CI 91.9%–96.7%) (p = 0.003). Molecular findings showed no artemisinin resistance–associated genotypes and major increases in genotypes associated with high sensitivity/efficacy for amodiaquine than before ASAQ was introduced. Thus, the efficacy of ASAQ is maintained and appears to be increased after long-term use in contrast to what is observed for other ACTs used in Africa.

Artemisinin-based combination therapy (ACT) has been first-line treatment for uncomplicated *Plasmodium falciparum* malaria globally for the past 10–15 years and has contributed greatly to a reduction of malaria illnesses and deaths during 2005–2015 (*1*,*2*). However, artemisinin resistance emerged in Cambodia during 2008, where it then spread and even developed de novo throughout the Great Mekong Region (*3*,*4*). Possible resistance has been reported from eastern India (*5*) and, Guyana in South America (*6*) but not yet from Africa (*4*). However, ACT resistance represents a continuous threat in contexts such as Zanzibar, where numerous long-distance visitors represent a special risk for imported artemisinin-resistant malaria parasites. Chloroquine resistance entered eastern Africa most probably from India in late 1970s (*7*). In addition, selection of resistance/tolerance to the slowly eliminated long-acting partner drugs in ACT (e.g., amodiaquine) is expected, especially in highly malaria-endemic areas of Africa (*8*–*10*), which could result in relatively reduced ACT cure rates and reduced protection against artemisinin resistance (*11*). Currently, however, complete ACT resistance has developed and spread only in Asia (e.g., Cambodia) (*12*).

In Zanzibar, malaria transmission has been reduced substantially after new and reinforced malaria tools and interventions, including ACT for uncomplicated malaria (*2*), were implemented. The reduced parasite biomass on the islands of Zanzibar has resulted in an expected selection (bottleneck) of the parasite populations (*2*,*13*), which under strong drug exposure might select for drug resistance. The first-line ACT in Zanzibar has been artesunate/amodiaquine (ASAQ) since 2003, plus recently added single, low-dose primaquine. Artemether/lumefantrine was used as second-line treatment when ACT was first used, followed by quinine when treatment guidelines were revised in 2009 (*2*). Free access throughout the health systems has resulted in sustained high population coverage and compliance to ASAQ (*2*,*14*,*15*). The partner drug amodiaquine is relatively short-lived (half-life 2–8 hours) and is primarily metabolized to its main biologically active metabolite desethyl-amodiaquine, which has a longer terminal elimination half-life (>7 days) (*16*).

Efficacy verses resistance to ACTs is primarily assessed by the in vivo response to standard treatment in which early clearance determines the effect of the artemisinin component, and the cure rate by days 28 or 42 after treatment determines the effect of the combination, especially that of the partner drug (*17*). Tolerance/resistance to the ACT components can also be estimated separately by genetic determination of different drug-resistance associated polymorphisms. A few longitudinal studies in Africa have examined tolerance/resistance trends to ACTs, especially to artemether/lumefantrine, suggesting largely maintained treatment efficacy but also higher prevalences of genotypes associated with tolerance to lumefantrine (*18*,*19*). However, there is a lack of combining longitudinal in vivo cure rates and molecular findings, particularly in relation to ASAQ, the second most widely used ACT in Africa.

In Zanzibar, 2 previous clinical trials in 2002–2003 (*20*) and 2005 (A. Bjorkman, unpub. data) showed high efficacy of ASAQ. A study of gene polymorphisms did not show any early trends of drug resistance selection after wide-scale ACT implementation (*21*). We conducted a new clinical trial and molecular survey of *P. falciparum* genes in 2017, after 14 years of large-scale use of ASAQ as first-line treatment. The objective of this study was to estimate the *P. falciparum* sensitivity to ASAQ, including both the in vivo treatment efficacy and the parasite profiles with regards to drug resistance–associated molecular characteristics (markers). The in vivo and molecular results were then compared with findings from previous studies conducted during 2002–2013.

## Materials and Methods

### Study Design and Area

**Figure 1:**
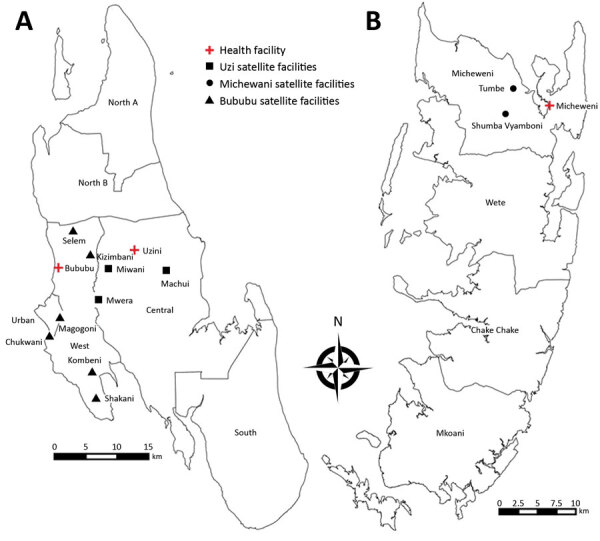
Locations of 14 study health centers, including 11 peripheral satellite health units and 3 referral health facilities for which increased sensitivity of *Plasmodium falciparum* to artesunate/amodiaquine despite 14 years as first-line malaria treatment was tested, Zanzibar. A) Unguja Island; B) Pemba Island.

We conducted a single-armed therapeutic efficacy trial of ASAQ (standard dose) and primaquine (single low-dose) treatment for uncomplicated *P. falciparum* malaria (ClinicalTrials.gov identification no. NCT03773536), in accordance with World Health Organization (*22*) and Worldwide Antimalarial Resistance Network guidelines (*23*), in the West and Central Districts (Unguja Island) and Micheweni District (Pemba Island) during May–September 2017. Study participants were recruited from 14 primary health-care units, including 11 peripheral satellite facilities and 3 referral health facilities (Figure 1). We selected the facilities on the basis of relatively high malaria detection rates in 3 preceding months and proximity to 3 centrally located referral centers in the respective districts. The study was implemented in accordance with the Helsinki Declaration and approved by the Zanzibar Medical Research Ethical Committee, Zanzibar Food and Drug Board, and Regional Research Ethics Board in Stockholm, Sweden.

### Study Participants

The participants (all ages) were recruited among febrile patients attending the 14 selected health facilities. They were screened by using malaria rapid diagnostic tests (mRDTs), and if positive results were found, they were referred to the closest referral center. Participants were considered eligible for study inclusion if they were confirmed to be febrile (axillary temperature >37.5°C) or had a history of fever (past 48 hours) and confirmed microscopically with any level of asexual *P. falciparum* parasitemia. They were finally enrolled if considered able to comply with the study protocol (e.g., residence <10 km from referral center) and if written informed consent was obtained from patient, parent, or guardian. Exclusion criteria were severe malaria signs, underlying disease, positive pregnancy test result, or suspected alternative reason for the febrile condition.

### Study Procedure

The enrolled patients were given treatment and followed-up at the referral centers. They received the antimalarial standard treatment orally (i.e., fixed-dose combination of artesunate [4 mg/kg] plus amodiaquine [10 mg/kg]: ASAQ Winthrop; Sanofi Pharmaceuticals, https://www.sanofi.com) once a day for 3 consecutive days. A single low-dose (0.25 mg/kg) of primaquine (primaquine phosphate; Sanofi Pharmaceuticals) was co-administered on the first day (D0). All doses were administered under direct supervision and observation for 30 min. If vomiting occurred, the patient was again given the same supervised dose and withdrawn from the study if a second vomiting occurred.

After the first 3 treatment days (D0, D1, and D2), the follow-up consisted of fixed appointments on D3, D7, D14, D21, and D28 and whenever the patient experienced any clinical symptoms. To ensure optimal compliance to the study protocol, an incentive of 5,000 Tanzanian shillings (US $2.20) was provided upon each visit, mainly to cover transportation costs.

At each follow-up visit, standard physical examination was performed and temperature was recorded. Finger-prick blood samples were collected for thick blood films and dried blood spots on filter paper (3MM; Whatman, https://www.cytivalifesciences.com). These filter papers were packaged in desiccated individual plastic bags and sent to the Karolinska Institutet (Stockholm, Sweden) for molecular analyses.

Microscopy reading of Giemsa-stained blood films was performed by 2 experienced microscopists at each visit. We quantified parasitemia by using the standard approximation method (40× parasites/200 leukocytes). A blood film was defined as positive if >1 asexual parasite was found per 1,000 leukocytes and the final parasite density was the average of 2 independent reads. An independent examination by a third microscopist was performed in case of discordant reads. We measured hemoglobin levels by using a HemoCue B-Hemoglobin Photometer (https://www.hemocue.com) on D0, D3, D7, D14, and D28, or any day of clinical suspicion of (hemolytic) anemia.

### Molecular Analyses

We conducted PCR screening for all filter paper blood spots collected on D0, D3, D7, and D28 after DNA was extracted by using the Chelex-boiling method (*21*). A quantitative PCR specific for the rRNA genes of *Plasmodium* species was used to screen for parasite DNA and estimate parasite densities (*21*). Samples with cycle values <40 in duplicate runs were considered parasite positive. Single-nucleotide polymorphisms (SNPs) in the *P. falciparum* multidrug-resistance 1 (*pfmdr1*) and *P. falciparum* chloroquine-resistance transporter (*pfcrt*) genes, associated with amodiaquine resistance (*8*,*9*) were analyzed in all D0 samples. SNPs at positions *pfcrt* K76T, *pfmdr1* N86Y, Y184F, and D1246Y were analyzed according to established nested PCR–restriction fragment length polymorphism protocols (*21*). In addition, polymorphisms in the 850-bp fragment of the *P. falciparum* Kelch13 propeller domain (*pfk13*), associated with artemisinin resistance were analyzed by using nested PCR amplification with Q5 high-fidelity polymerase (New England Biolabs, https://www.neb.uk.com), followed by direct Sanger sequencing of the PCR amplicon (*24*).

### Study Outcomes

The primary outcome was PCR-corrected treatment failure rates assessed after 28 days. Secondary outcomes were parasite and fever clearance rates by D3, hemoglobin decrease by D7, residual PCR positivity, and D0 genotype profiles associated with parasite tolerance/resistance.

### Comparator Studies

We compared the current clinical trial with 2 previous in vivo trials conducted in Zanzibar during 2002–2003 (*20*) and 2005 (A. Bjorkman, unpub. data) (ClinicalTrials.gov identifiers NCT03764527 and NCT03768908). Both studies were open-label, randomized, 2-armed studies comparing in vivo efficacy of ASAQ and artemether/lumefantrine in children (<5 years of age) with uncomplicated *P. falciparum* malaria (range 2,000–20,0000 parasites/μL). Study procedures were similar to those in 2017. We performed paired PCR genotyping of the *P. falciparum* merozoite surface protein 2 gene in samples collected on D0 and for recurrent parasitemia days 14–28 (*20*) to differentiate reinfection from recrudescence.

We compared prevalences of molecular markers of drug resistance during 2002–2003, 2005, and 2017, as well as published data for 2010 and 2013 (*21*). Molecular genotyping of SNPs was conducted by using the same protocols (*21*) in all studies. *Pfk13* sequencing was only conducted in samples from 2017.

### Samples Size

Sample size for the 2017 clinical trial was calculated for an estimated efficacy rate of 95% and a 95% CI within a total width of 10%. To achieve this power, 90 patients were required after attrition losses estimated to be 20%. However, we targeted 150 patients, a comparable number to those of previous trials (2002–2003 and 2005).

### Statistical Analyses

We entered data into Microsoft Excel (https://www.microsoft.com) and cleaned data by using GSPro (https://www.dji.com). We performed statistical analyses by using Stata (https://www.stata.com). We calculated 95% CIs for proportions of patients cured by D28 by using the exact method described by Fagan (*25*); we compared proportions by using the Fisher exact test. We assessed associations between PCR positivity and patient characteristics at study baseline by using the Fisher exact test or Wilcoxon rank-sum (Mann-Whitney) test. We conducted trend analyses for genotypes by using logistic regression and year as a continuous variable. We performed analysis for the proportion of patients harboring mutant alleles (i.e., only mutant, or mixed with wild-type), as well as for the ratio of infections with mutants versus infections with the corresponding wild-types (mutants *pfcrt* 76T; *pfmdr1* 86Y, Y184, and 1246Y).

## Results

### Patients

The 14 health centers screened 9,062 febrile patients during May–September 2017; a total of 233 (2.6%) were positive by mRDT, and 146 satisfied all inclusion criteria and thus enrolled at the 3 referral centers. We provide demographic, clinical, and laboratory characteristics for patients (Table 1), along with data from the previous clinical trials during 2002–2003 and 2005. Despite different inclusion criteria regarding age and parasitemia, the geometrical mean parasite densities and ranges at study enrollment were similar (Table 2). A total of 142 (97%) of 146 patients in 2017 completed the study follow-up to D28. Four patients were excluded or did not complete follow-up because of vomiting on D1, itching on D3, too long travel distance to a referral center, and travel to mainland Tanzania.

**Table 1:** Characteristics of patients in 3 clinical trials testing increased sensitivity of *Plasmodium falciparum* to artesunate/amodiaquine despite 14 years as first-line malaria treatment, Zanzibar

Characteristic	Study group		
	2002–2003	2005	2017
No. screened*	2,097	2,076	9,062
No. enrolled	207	177	146
M:F ratio	104:103	82:95	101:45
Median age (range)	24 (5–73) mo	28 (4–60) mo	16 (2–60) y
Geometric mean parasite density per microliter (range)	19,731 (2,000–198,000)	20,890 (2,000–176,000)	7,886 (75–304,000)
Mean ± SD temperature, °C	38.7 ± 1.2	37.8 ± 1.2	37.8 ± 1.4
Mean ± SD hemoglobin level, g/dL	8.5 ± 1.6	9.2 ± 1.4	11.9 ± 2.2

*Febrile patients attending healthcare facilities with suspected uncomplicated *P. falciparum* malaria infections.

**Table 2:** Characteristics of patients fulfilling study protocol in 2017, by age group and parasite density, Zanzibar

Characteristic	Study group				
	2017, all patients	2017, children <5 y of age	2017, children <10 y of age	2017, children <15 y of age	2017, parasite density >2,000/μL
Total	142	21	42	66	115
M:F ratio	99:43	13:8	28:14	45:21	81:34
Median age (range)	17 (2–60) y	48 (21–60) mo	5.5 (1.8–10) y	9 (1.8–15) y	16 (1.8–60) y
Geometric mean parasite density/μL (range)	7,899 (75–304,000)	15,773 (85–304,000)	11,847 (85–304,000)	10,618 (85–304,000)	14,305 (2,175–304,000)
Mean ± SD temperature, °C	37.8 ± 1.4	38.2 ± 1.2	38.1 ± 1.1	38.2 ± 1.2	37.9 ± 1.4
Mean ± SD hemoglobin level, g/dL	11.9 ± 2.2	9.1 ± 1.7	9.8 ± 1.9	10.6 ± 2.1	11.8 ± 2.3

### Treatment Outcomes

**Figure 2:**
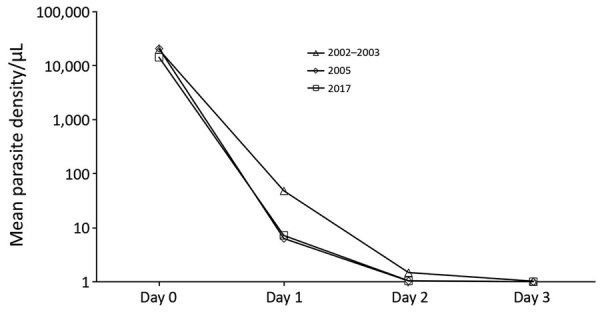
Comparison of parasite clearance rates until day 3 posttreatment for 3 study periods, Zanzibar. Microscopy determined geometrical mean parasite densities. Only parasite densities >2,000 parasites/μL on day 0 were included in 2017. Microscopy negative samples were given an arbitrary value of 1 parasite/μL.

Parasite clearance rates by microscopy up to D3 were similar in the 3 trials (Figure 2; Table 3). In 2017, one patient remained malaria positive at D3, after which all were microscopy negative up to D28 (Table 4). The cure rate of 100% (95% CI 97.4%–100.0%) in 2017 was higher when compared with the PCR-adjusted cure rate for 2002–2003 and 2005 combined (358/378, 94.7% [95% CI 91.9%–96.7%]; p = 0.003). Statistical significance was maintained after including only patients with >2,000 parasites/μL on day 0 in 2017 (p = 0.006), and near significance (p = 0.055) was achieved when adjusting for age <15 years (i.e., with <5 years of exposure to major malaria transmission. Numbers of recurrent parasitemias, defined as new infections during the 28 days of follow-up, were 44 (22%) in 2002–2003, 16 (9%) in 2005, and none (0%) in 2017, confirming higher malaria transmission rates in 2002–2005.

**Table 3:** Parasite clearance determined by microscopy in *Plasmodium falciparum*–positive persons, Zanzibar*

Characteristic	Study group				
	2002–2003, n = 206	2005, n = 172	2017, all patients, n = 142	2017, age <5 y, n = 21	2017, parasite density >2,000/μL, n = 115
Parasite positivity by microscopy, no.; % (95% CI)					
Day 0	206; 100 (98–100)	172; 100 (98–100)	142; 100 (97–100)	21; 100 (84–100)	115; 100 (97–100)
Day 1	137; 67 (60–73)	64; 37 (30–45)	58; 41 (33–49)	12; 41 (34–78)	53; 46 (37–56)
Day 2	18; 9 (5–13)	1; 0.6 (0–3)	2; 1 (0–5)	0; 0 (0–16)	1; 1 (0–5)
Day 3	1; 0.5 (0–3)	0; 0 (0–2)	1; 0.7 (0–4)	0; 0 (0–16)	0; 0 (0–3)
Geometric mean parasite density/µL for parasite-positive persons (range)					
Day 0	19,858 (2,000–198,447)	20,822 (2,000–176,000)	7,899 (75–304,000)	15,773 (85–304,000)	14,305 (2,175–304,000)
Day 1	359 (9–173,882)	397 (12–25,000)	822 (30–13,700)	802 (64–4,970)	83 (30–13,700)
Day 2	89 (32–552)	560, NA	500 (250–1,000)	NA, NA	1,000, NA
Day 3	78, NA	NA, NA	120, NA	NA, NA	NA, NA

*Values are proportions of patients positive by microscopy and with geometric mean parasite densities on days 0, 1, 2, and 3 after treatment. NA, not applicable.

**Table 4:** Artesunate/amodiaquine treatment outcome with 28-day follow-up for increased sensitivity of *Plasmodium falciparum* to artesunate/amodiaquine despite 14 years as first-line malaria treatment, Zanzibar

Year of study, group	No patients*	No. (%) positive on day 3	No. (%) with parasite recrudescence†	No. (%) with recurrent new infection	p value‡
2002–2003, all patients§	206	1 (0.5)	13 (6)	44 (22)	Referent
2005, all patients§	172	0	7 (4)	16 (9)	Referent
2017, all patients	142	1 (1)	0	0	0.003
2017, children <5 y of age	21	0	0	0	0.614
2017, children <10 y of age	42	0	0	0	0.243
2017, children <15 y of age	66	0	0	0	0.055
2017, >2,000 parasites/µL	115	0	0	0	0.006

*Number fulfilling follow-up as per protocol. †After PCR correction. ‡p value when compared with 2002–2003 and 2005 combined. §All patients were ≤5 y of age and had parasite densities >2,000 parasites/µL in 2002–2003 and 2005.

PCR positivity and parasite density estimates by quantitative PCR were analyzed only for the 2017 study (Table 5). Patients remained positive for much longer by PCR than by microscopy. PCR positivity on D3 and D7 were strongly associated with age, parasite density at study enrollment, baseline temperature, and hemoglobin value (Table 6). Associations were not significant for persons who were PCR positive on D28.

**Table 5:** Parasite clearance determined by qPCR after treatment with ASAQ and single, low-dose primaquine, Zanzibar, 2017*

Day after treatment	Parasite positivity by PCR, no.; % (95% CI)	qPCR-determined geometric mean parasite density/μL (range)
Day 3	90; 63 (55–71)	2 (<1−796)
Day 7	42; 30 (22–37)	<1 (<1−18)
Day 28	9; 6 (2–10)	1 (<1−58)

*ASAQ, artesunate/amodiaquine; qPCR, quantitative PCR.

**Table 6:** Association between PCR positivity on days 3, 7, and 28 after treatment and patient characteristics at study baseline, Zanzibar*

Characteristic	PCR negative, day 3	PCR positive, day 3	p value,† day 3	PCR negative, day 7	PCR positive, day 7	p value,† day 7	PCR negative, day 28	PCR positive, day 28	p value,† day 28
Total (%)	52/142 (37)	90/142 (63)	NA	100/142 (70)	42/142 (30)	NA	133/142 (94)	9/142 (6)	
M:F ratio	33:19	65:25	0.35	70:30	28:14	0.70	91:42	72	0.72
Median age, y (range)	24 (2–57)	14 (2–60)	<0.001	19 (2–60)	12.5 (2–54)	0.01	17 (2–60)	14 (9–56)	0.54
Geometric mean parasite density/µL (range)	3,998 (75–120,955)	12,011 (78–304,269)	<0.001	6,185 (76–145,750)	14,940 (561–304,269)	0.01	7,847 (78–304,269)	11,259 (2,730–56,304)	0.61
Mean ± SD temperature, °C	37.2 ± 1.2	38.1 ± 1.3	<0.001	37.7 ± 1.4	38.0 ± 1.2	0.27	37.8 ± 1.4	38.0 ± 1.2	0.62
Mean ± SD hemoglobin level, g/dL	12.4 ± 2.2	11.7 ± 2.2	0.09	12.2 ± 2.2	11.3 ± 2.2	0.01	12.0 ± 2.3	11.4 ± 1.5	0.37

*NA, not applicable. †Calculated by Fisher exact test (for sex) or Wilcoxon rank-sum (Mann-Whitney) test for continuous variables.

Fever clearance rates were similar: temperatures <37.5°C by D3 in 92.8% (95% CI 88.4%–95.9%) of patients in 2002–2003, 98.8% (95% CI 95.9%–99.9%) of patients in 2005, and 97.9% (95% CI 94.0%–99.6%) of patients in 2017. Hemoglobin levels at enrollment (D0) were higher in all patients in 2017 (Table 1) but similar in children <5 years of age when compared with 2002–2003 and 2005 (Table 2). The average decrease by D7 was −1.10 g/dL (range −6.1 g/dL to –3.3 g/dL) in 2017, when primaquine was added to ASAQ, and −0.20 g/dL (range −3.6 g/dL to –3.4 g/dL) in 2002–2003 after ASAQ alone (p<0.001). However, after adjusting for hemoglobin level at D0, the decrease was more pronounced in 2002–2003 (−0.60 g/dL) than in 2017 (−0.09 g/dL) (p = 0.003). There was no case of severe anemia (hemoglobin level <5 g/dL) at D0 or D7 in any study. No patient experienced any serious adverse event.

### Polymorphisms in *pfcrt*, *pfmdr1*, and *pfk13* Genes

**Figure 3:**
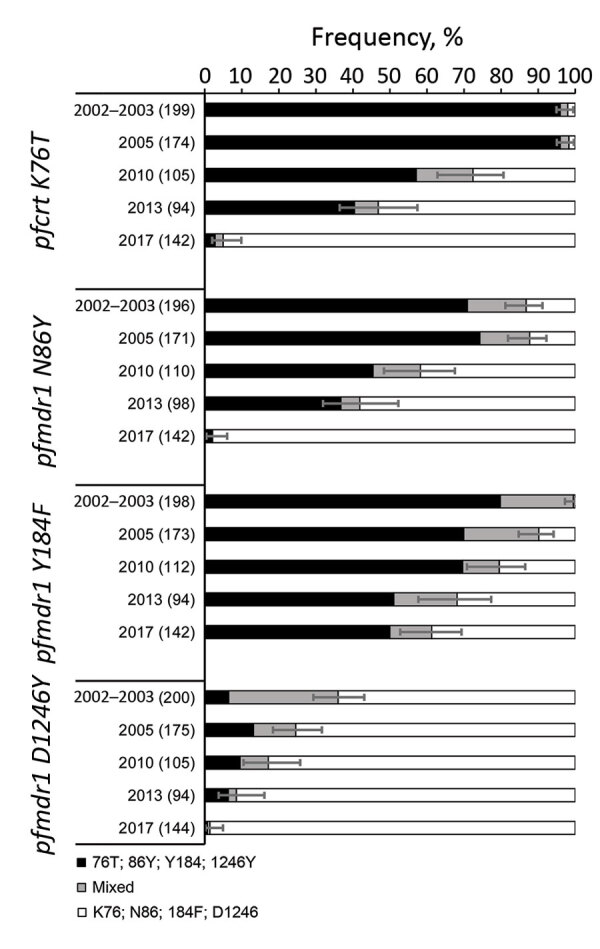
Frequency of polymorphisms associated with amodiaquine resistance in *Plasmodium falciparum* infections in Zanzibar, 2002–2017. Black bars indicate resistance alleles, gray bars indicate mixed infections, and white bars indicate wild-type alleles. Error bars indicate 95% CIs of proportions of infections harboring resistance alleles (either alone or mixed infections). Values in parentheses are the total number of genotyped samples shown next to the study year. Trend analysis: p<0.001 for *pfcrt* 76T + mixed, *pfmdr1* 86Y + mixed, *pfmdr1* Y184 + mixed, *pfmdr1* 1246Y + mixed; p<0.001 for *pfcrt* 76T, *pfmdr1* 86Y, *pfmdr1* Y184Y; and p = 0.016 for *pfmdr1* 1246Y. Pfcrt, *P. falciparum* chloroquine-resistance transporter gene; Pfmdr1, *P. falciparum* multidrug-resistance gene.

There was a significant reduction of *pfcrt* K76T prevalence from 98.0% in 2003 to 4.9% in 2017 (p<0.001) (Figure 3) and negative trends were also seen for *pfmdr1* 86Y, Y184, and 1246Y, all associated with reduced sensitivity to amodiaquine (*8*,*9*,*26*). *Pfmdr1* YYY and YYD were the most frequent haplotypes in 2002–2003, and the NYD and NFD haplotypes were most frequent and the YYY totally absent in 2017 (Table 7). The decrease of YYY and YYD was highly significant between 2002–2003 and 2017 (p<0.001).

**Table 7:** *Plasmodium falciparum* multidrug-resistance 1 haplotype frequencies in clinical trials of increased sensitivity to artesunate/amodiaquine despite 14 years as first-line malaria treatment, Zanzibar*

Haplotype	Study group					p value†	p value‡
	2002–2003, n = 161	2005, n = 156	2010, n = 92	2013, n = 87	2017, n = 140		
YYY	31.1	20.5	12.0	8.0	0.0	<0.001	<0.001
YYD	57.1	63.5	38.0	31.0	2.1	<0.001	<0.001
YFD	0.0	4.5	7.6	0.0	0.0	<0.001	NA
NYD	11.2	7.1	26.1	27.6	58.6	<0.001	<0.001
NFY	0.0	0.0	0.0	1.1	0.7	0.25	0.46
NFD	0.6	4.5	16.3	32.2	38.6	<0.001	<0.001

*Values are percentages unless indicate otherwise. NA, not applicable. †Comparing frequencies between all years by Fisher exact test. ‡Comparing frequencies between 2002–2003 and 2017 by Fisher exact test.

Regarding the *pfK13* gene, 139 (98%) of 142 samples collected 2017 were successfully sequenced. No nonsynonymous SNP was identified. Two synonymous mutations were found, the SNPs C469C (5 samples) and S477S (1 sample). Five samples were from patients at the Bububu health center, and 1 (C469C) was from a patient in Uzini who had traveled to mainland Tanzania.

## Discussion

The high cure rate in 2017 (100%) was significantly higher than the combined cure rate in 2002–2003 and 2005 (94.7%) (p = 0.003). The microscopy-determined parasite clearance was as rapid as that in 2002–2003 and 2005 (<3 days), and fever clearance was similar. These findings confirm maintained full efficacy of the artesunate compound (*17*) and suggest an increased cure rate by ASAQ. Compliance to the study protocol was high: only 4/146 patients were unable to complete follow-up despite logistical constraints of conducting the clinical trial in the low-transmission context in 2017. A large enough sample size was achieved by recruiting patients from 11 peripheral satellite health units and 3 referral health facilities (in which follow-up attendances were conducted) and by including patients of all ages and all parasite densities.

High in vivo efficacy is in agreement with previous findings from Madagascar (*27*) and Côte d’Ivoire (*28*) after 6 and 10 years of ASAQ use as first-line treatment, respectively. It might be argued that the observed higher cure rate in 2017 may be caused by a different study age group; added single low-dose primaquine; fixed-dose versus a loose combination of ASAQ compounds; or reduced malaria transmission compared with that in 2002–2003 and 2005. However, age-related protective immunity in the population in Zanzibar has decreased substantially (*2*) and thus is expected to have had little influence on cure rate, especially in children <15 years of age. Single low-dose primaquine is not expected to have had any major effect on the asexual *P. falciparum* stage (*29*), and whereas different efficacies by different ASAQ formulations have been reported in a large meta-analysis (*30*), no significant difference was found between a nonfixed loose combination of ASAQ with an amodiaquine dose of 30 mg/kg (used in the studies during 2002–2003 and 2005) compared with a fixed combination with same amodiaquine dose (used in our 2017 study). In addition, drug intake with the loose combination was highly supervised during 2002–2003 and 2005; some new infections might have been falsely misinterpreted as recrudescent during higher transmission in 2002–2003 and 2005, the opposite might also occur (*31*–*33*). A potential general limitation in the in vivo assessment of ASAQ efficacy, although according to World Health Organization recommendations (*22*), is that a 28-day follow-up might miss some late recrudescences (*20*). However, this possibility should not affect the comparative approach.

A minor reduction of hemoglobin levels from D0 to D7 was similarly observed in 2017 and 2002–2003 and 2005 and is consistent with common findings after ACT treatment (*34*). This reduction supports the safety of adding single low-dose primaquine in 2017 despite ≈10% prevalence of glucose-6-phosphate dehydrongenase deficiency in the population in Zanzibar.

Residual parasite positivity by PCR on D3 and several weeks posttreatment despite observed high ACT efficacy has been described and does not necessarily imply drug resistance (*35*–*37*). A key factor associated to such positivity was, as expected, high initial parasite density. What low-grade PCR positivity represents remains unclear, be it residual parasite DNA, gametocytes, or suppressed dormant and potentially surviving parasites (*37*,*38*).

Our study showed no sign of increased tolerance to artesunate because no resistance-associated mutations were detected in the *pfk13* gene, in accordance with several other studies in Africa (*4*). Moreover, prevalences of the SNPs *pfcrt* 76T and *pfmdr1* 86Y, Y184, and 1246Y associated with amodiaquine resistance (*8*,*9*,*26*) all decreased steadily during the observation period (Figure 3). Amodiaquine and chloroquine do generally select for similar mutations in the *Pfcrt* and *Pfmdr* genes. However, whereas chloroquine, used as first-line treatment up to 2003, did strongly select such mutations, this finding was reversed when amodiaquine combined with artesunate became first-line treatment. This finding is quite in contrast to longitudinal studies in areas that used other ACTs as first-line treatment. *P. falciparum* genotypes associated with tolerance/resistance to the ACT partner drugs lumefantrine (*18*,*39*), sulfadoxine/pyrimethamine (*40*,*41*) and piperaquine (*12*) have all been shown to consistently increase over time, after respective ACT use.

A major objective for combination therapy is for combined compounds to protect each other (i.e., preventing selection of resistance to either drug and both drugs combined). In Zanzibar, our in vivo and molecular findings suggest that *P. falciparum* has become increasingly sensitive to the combination ASAQ, despite its widescale use since 2003–2004. Although changes in allele frequencies in Zanzibar could be caused by genetic drift after rapid reduction in the parasite population in Zanzibar causing a genetic bottleneck, the temporal trends of *Pfcrt* and *Pfmdr1* alleles suggest a selection event as more likely. However, why are *pfcrt* and *pfmdr1* mutations associated with amodiaquine resistance selected against, in favor of the drug-sensitive wild-types over time despite being temporarily selected after each ASAQ treatment (*8*,*9*)?

Resistance mutations confer an advantage in the presence of the drug, although such mutations often come with fitness costs in the absence of the drug (*42*–*44*) (sometimes additional compensating mutations might also restore fitness in mutated parasites [*45*]). Thus, spread of drug resistance alleles might mostly be restricted when in competition with wild-type parasites in the absence of the drug (*46*,*47*). Such competition is expected in contexts such as Zanzibar, where decreasing transmission rates have led to a corresponding decrease in ASAQ use over time, especially because treatment is normally restricted to mRDT-positive patients only (*15*). In addition, a large proportion of infections are subpatent and thus represent a large reservoir of competing parasites unexposed to antimalarial drugs (*2*).

Potentially contributing to increased sensitivity to amodiaquine are infections imported from mainland Tanzania (*2*,*48*), where the first-line treatment is artemether/lumefantrine, which selects the opposite genotypes of those for amodiaquine (*8*–*10*). Another potential reason for less resistance selection by amodiaquine compared with lumefantrine might be different pharmacokinetic profiles and thus different selection windows after treatment with the respective ACTs. However, the immediate selection dynamics posttreatment have appeared rather similar for amodiaquine and lumefantrine (*8*–*10*). Accordingly, elimination kinetics of desethyl-amodiaquine, (elimination half-life initially short but terminally >7 days) (*16*) and lumefantrine (elimination half-life ≈3 days) (*49*) are not highly different.

In areas of increased resistance to partner drugs, ACT cure rates have mostly remained relatively high as long as artemisinins remained highly effective (*11*), except for artesunate–sulfadoxine/pyrimethamine (*40*,*41*). When artemisinins were still effective in Southeast Asia, selection of mefloquine resistance after previous monotherapy was initially stopped and temporarily reversed when the ACT artesunate/mefloquine was introduced (*50*). However, reduced partner drug efficacy will always represent increased risk for development and selection of artemisinin resistance. When artemisinin starts to fail in addition to the failing partner drug, an accelerating and alarming development toward multidrug resistance to the combination is expected. This suggestion has occurred in the Greater Mekong Subregion for dihydroartemisinin/piperaquine (*12*).

Despite 14 years of widescale use of ASAQ as a first-line treatment for malaria in Zanzibar, there are no indications of increased tolerance/resistance to either drug. Our in vivo and molecular findings suggest an increased antimalarial activity by the partner drug amodiaquine. We believe that this finding might be primarily caused by fitness costs of the amodiaquine tolerance/resistance–related mutations in the *pfcrt* and *pfmdr1* genes in the low-transmission context with restricted and compliant use of ASAQ to parasitologically confirmed malaria cases only, and with relatively frequently imported parasites without chloroquine/amodiaquine resistance–associated mutations. ASAQ might have a comparative advantage, especially in low-transmission areas compared with other ACTs against development or spread of artemisinin- (and ACT-) resistant parasites.
